# The physical and developmental outcomes of children whose mothers are substance abusers: Analysis of associated factors and the impact of early intervention

**DOI:** 10.3389/fped.2022.1004890

**Published:** 2022-10-20

**Authors:** Anna Wai Fun Cheng, Hin Biu Chan, Lai Sheung Ip, Katy Kit Ying Wan, Ellen Lok Man Yu, Wa Keung Chiu, Pui Hong Chung, Eng Kiong Yeoh

**Affiliations:** ^1^Department of Pediatrics and Adolescent Medicine, United Christian Hospital, Hong Kong, Hong Kong SAR, China; ^2^Department of Obstetrics and Gynaecology, United Christian Hospital, Hong Kong, Hong Kong SAR, China; ^3^Rainbow Lutheran Centre, Hong Kong Lutheran Social Service, Hong Kong, Hong Kong SAR, China; ^4^Clinical Research Centre, Kowloon West Cluster, Hospital Authority, Hong Kong, Hong Kong SAR, China; ^5^Centre for Health Systems and Policy Research, The Jockey Club School of Public Health and Primary Care, Faculty of Medicine, The Chinese University of Hong Kong, Hong Kong, Hong Kong SAR, China

**Keywords:** maternal substance abuse, physical health, developmental outcome, 0 to 5 years old children, early integrated program, multidisciplinary collaboration, social support

## Abstract

**Background/objectives:**

Maternal illicit drug use is associated with negative physical and developmental outcomes for their born children. We aim to find out the incidence of different developmental problems in a cohort of Chinese children born to drug-abusing mothers, compare the physical health and developmental outcomes of the subjects recruited in the Integrated Program to the Comprehensive Child Development Service (CCDS), and to study the potential factors on their associations.

**Methods:**

A retrospective longitudinal cohort study with frequent clinical assessments of the children’s physical and developmental outcomes in a HKSAR’s regional hospital from birth until 5 years old. 123 Children in Integrated Program were compared with 214 children in CCDS between 1 January 2008 and 28 February 2019. Cox regression analysis was performed to determine the possible factors associated with the developmental outcomes.

**Results:**

Developmental delay was detected in 129 children (38.9%). CCDS group has significantly higher incidence of cognitive delay (*p* = < 0.001), language delay (*p* = < 0.001), motor delay (*p* = < 0.001), social delay (*p* = 0.002), and global delay (*p* = 0.002). On Cox multivariable regression analysis, integrated program (HRadj 0.53, 95% C. I. 0.34–0.84), social support (HRadj 0.45, 95% C.I. 0.25–0.80), and maternal abstinence from drug use up to 2-year post-delivery (HRadj 0.62, 95% C.I. 0.40–0.95) were significant protective factors, while male gender (HRadj 1.73, 95% C.I. 1.18–2.54) was a significant risk factor.

**Conclusion:**

CCDS achieves early engagement of drug-abusing expectant mothers during pregnancy, and an early integrated program with multidisciplinary collaboration was an independent factor in improving the developmental outcomes of these vulnerable children.

## Introduction

Maternal substance abuse is a long-standing and significant ongoing problem that poses great public health concern and is associated with negative outcomes for both the mother and her child (1–4). Drugs can affect normal growth and alter the neurotransmitters in the fetal brain, e.g., cocaine has effects on cortical neuronal development (5, 6). Methamphetamine can alter neurotransmitters in the brain and affects brain morphogenesis (7, 8), an increased risk for neuronal development and connectivity causing subsequent neurobehavioral deficit (9).

Dose-related motor deficits had been reported in children with prenatal cocaine exposure (10–12). Various studies have found impaired language development and specific receptive language difficulties (13–16) in children whose mother with a history of cocaine addiction. However, another study found no association of prenatal exposure to cocaine and opiates with mental or motor deficits after controlling for birth weight and other environmental risk factors (17). Children exposed to drug use during pregnancy had more problems related to cognitive and social development than other children (18). Most studies reported that the negative impacts on cognitive development in infants and pre-schoolers were related to prenatal exposure (19–22), whereas others stressed the deprived socioeconomic background as the main contributing factor (23–25).

Some earlier studies have shown difficulties in language development in these children born to mothers with substance use (21, 23, 25). Continuing drug use by mothers of drug-exposed children predicted poor cognitive development in the school years (26, 27). Parental substance abuse was related to disorders of psychological and behavioral development in children (28–30), mental health problems, and substance use in adolescence and adulthood (31–33). Carers’ parenting stress and psychiatric symptoms were also associated with children’s emotional and behavioral outcomes (34).

Most of these studies on child developmental outcomes were not done with professional follow-ups and assessments. Comprehensive Child Development Service (CCDS) in collaboration with the Counselling Centre for Psychotropic Substance Abusers in Kowloon East Cluster (KEC) aims to improve the physical and developmental outcomes of the children born to drug-abusing mothers. KEC is one of the seven clusters of the Hospital Authority in Hong Kong SAR, China. With intersectoral and multidisciplinary collaboration and support, mothers can abstain from drug use, establish their role as good-enough parents, and provide a nurturing environment to raise their children as women who continue to use substances after delivery were unable to do so (35, 36). We employ a longitudinal study using clinical samples with professional diagnosis, a less-stigmatizing platform where more than 90% of children born to the general population would attend for routine health surveillance and childhood immunization from birth to 5 years old (Maternal and Child Health Centre) with frequent clinical assessments of subjects with the aim (1) to find out the incidence of different developmental problems in a cohort of Chinese children of substance-abusing mothers, (2) to compare the physical and developmental outcomes of children born to substance-abusing mothers recruited in the integrated program to the children born to substance-abusing mothers managed in CCDS, and (3) to study the potential parental factors and children’s individual factors affecting the association between maternal substance abuse and child developmental outcomes.

## Method

### Design

A retrospective cohort study from 1 January 2008 to 28 February 2019, in which children born to mothers with illicit soft drug use within 1 year of the expected date of delivery in a tertiary hospital in Hong Kong Kowloon East Cluster, was included. The group of children (*N* = 123) recruited in the integrated program was compared with the CCDS group (*N* = 214). Both groups were recruited *via* the Comprehensive Child Development Service Registry database which included the information on maternal illicit drug use, the demographics, and socioeconomic factors of both parents, and these data were retrieved and analyzed. The health and development of the recruited child was assessed every 2 to 6 months from birth until 5 years of age. Diagnosis and case notes of recruited children and mothers were reviewed by the principal investigator using the Hospital Authority Clinical Management System and Electronic Patient Record System. The occurrence of children and maternal outcomes at any time point during the follow-up were retrieved and recorded. Clinical diagnosis was either confirmed by the same CCDS Paediatrician or Child Assessment Centre, Child Psychiatrist or Clinical Psychologist. Non-Chinese mothers and children with gestation below 28 weeks, and known chromosomal, genetic, or metabolic diseases related to developmental delay were excluded. Ethics approval was obtained from the local Research Ethics Committee. Sample size calculation included in the Supplementary material.

### Comprehensive child development service since 2007 and integrated program since 2010 in Kowloon East Cluster, Hospital Authority, HKSAR, China

Comprehensive Child Development Service (CCDS) is a government-funded and community-based service aiming at early identification of children and families in need and integrating the medical and health, social, and education service sectors to provide timely intervention to vulnerable families. A multidisciplinary service involves the departments of Paediatrics and Adolescent; Obstetrics and Gynaecology and Psychiatry in Kowloon East Cluster of Hospital Authority, Maternal and Child Health Centre of the Department of Health, and various Integrated Family Service Units, Family and Child Protection Service Units of the Social Welfare Department, HKSAR, China.

Expectant mothers who have been using an illicit drug within 1 year of the expected date of delivery are engaged in the antenatal period by CCDS midwife with the education on nutritional needs, hygiene, and preparations of the baby delivery and discussion on the options of childcare and detoxification plan. The psychiatric team of CCDS offers ongoing assessment and treatment to mothers with psychiatric co-morbidity. A welfare meeting is arranged before the discharge of the newborn to formulate a comprehensive childcare and detoxification plan with the parent(s).

The growth and development of the born child are continuously assessed by the visiting CCDS pediatrician during the child’s health and vaccination sessions in the Maternal and Child Health Centre (MCHC). The child receives regular follow-up every 2 to 6 months until the age of 5, during which the mother is counseled on childcare techniques, handling of the child’s emotion and behavioral issues with anticipatory guidance to promote the child’s development, and good mother–child bonding.

For integrated program, expectant mothers were recruited by a CCDS midwife and referred to the Hong Kong Lutheran Social Service with their consent after a detailed explanation of the program. Apart from the usual service offered by CCDS, CCDS collaborated with Hong Kong Lutheran Social Service (HKLSS) with the emphasis on parent drug detoxification, parent education groups, parenting coaching, and parent support network for pregnant women with illicit drug use in addition to the core service offered by CCDS. Social workers of Hong Kong Lutheran Social Service accompany the expectant mother to have regular health visits at the tertiary hospital during the antenatal periods so as to ensure regular antenatal follow-up; accompany the mother and child to have regular visits at MCHCs to ensure meeting CCDS pediatrician and the child to receive immunization at the scheduled time, counsel, and motivate mother for drug detoxification and stop maternal high-risk behaviors like smoking and drinking, provide regular home visitations for emotional and parental support to the mother. Postnatal confinement service is also offered to the mother by the doula of HKLSS during the early months after delivery. Peer support classes for drug detoxification and parenting class are also held for mothers at HKLSS centers. Urine screening for toxicology is performed as planned with the mothers. There is a regular case review meeting held every 4 months to monitor the family by CCDS midwife and pediatrician, and HKLSS social workers so that maternal emotional or family issues can be identified with timely referral to the CCDS Psychiatrist or Integrated Family Social Service respectively for timely management; parenting and child developmental issues are also discussed with appropriate advice given by CCDS pediatrician for further enhancement of actions by HKLSS social workers.

### Measures

1. Children’s outcomes
a. Preterm as < 37 weeks of gestation at delivery.
b. Physical growth
  b1. Newborn period
      Low birth weight (LBW) as birth weight < 2,500 g
      Small for gestational age (SGA) as birth weight < 10th percentile for gestational age of the same sex in local population.
  b2. Childhood period
      Childhood growth problem defines as falling off 2 or more centiles from the growth curve of the same sex and age in local population.
c. Significant congenital malformations affecting organ function.
d. Delayed vaccination: the scheduled vaccination by the local Department of Health is delayed for more than 3 months without significant reason(s).
e. *Developmental delay by clinical assessment with reference to the developmental milestones
f. *Specific learning disorder, e.g., dyslexia
g. *Psychological and behavioral outcomes: attention-deficit hyperactive disorder (ADHD), autistic spectrum disorder (ASD), and non-specific behavioral disorders, emotional disorders.

*Developmental delay, specific learning disorder, psychological and behavioral outcomes

Developmental delay in cognitive, motor, speech, social aspects; emotional, behavioral, and learning problems is diagnosed by the same pediatrician at follow-up who has experience in Clinical Paediatrics or by formal assessment at the Child Assessment Centre or diagnosed by Child Psychiatrist or Clinical Psychologist in United Christian Hospital, Hong Kong SAR, China. The respective incidence of delay in cognitive, motor, speech and social aspects, behavioral, and learning problems is counted whenever it appears in the trajectory of follow-up. Any developmental delay is defined as a delay in one area with respect to cognitive, language, motor, or social development in the same child; two delays mean any combination of two areas in the child; and three delays mean any combination of three areas, and delays in all four areas are defined as a global delay in the child.

h. Child abuse/neglect is established after a multidisciplinary case conference held by different disciplines including medical professionals and social workers.
2. Maternal outcomes:
a. Maternal abstinence from drug use during pregnancy
b. Maternal relapse of drug use after birth
c. Maternal abstinence from drug use up to 2-year post-delivery

### Statistical analysis

IBM SPSS, version 26.0 (Armonk, NY: IBM Corp) was used for statistical analysis. Pearson’s chi-square and Fisher’s exact tests were used to compare categorical variables between integrated and CCDS groups. Independent *t*-test was used to compare numerical variables. Kaplan–Meier curve with log-rank test was used to compare the developmental delay between the two groups. Cox regression analyses (univariable and multivariable) were used to analyze the demographic and socioeconomic factors for adverse children’s outcomes. Variables with *p* < 0.1 in the univariable analysis were further assessed using multivariable analysis. The effect size was measured by hazard ratio. Associations between maternal outcomes and children’s outcomes were also evaluated. A two-sided *p*-value of less than 0.05 was considered significant.

## Results

A total of 360 cases, 128 from the integrated group and 232 from the CCDS group were recruited within the studied period. Five cases (3.9%) were excluded from the integrated group as three defaulted and two transferred out. Eighteen cases (7.8%) were excluded from the CCDS group as eight defaulted, nine transferred out, and one child suffered from creatine transport deficiency associated with global delay was also excluded.

The baseline characteristics between the integrated program group and CCDS group were mostly comparable, except there were significantly more boys in the CCDS group; maternal drug use duration was significantly shorter (4 vs. 4.7 years), and significantly more involvement of other adults in child-caring (76.6 vs. 59.3%) and significantly more children had been cared in out-of-home placement (14.5 vs. 5.7%) in the CCDS group than in the integrated program group (Table 1).

**TABLE 1:** Baseline characteristics (*n* = 337).

	Total	CCDS	Integrated	*P*-value
	(*n* = 337)	(*n* = 214)	(*n* = 123)	
**Sex**				0.025
Male	175 (51.9)	121 (56.5)	54 (43.9)	
Female	162 (48.1)	93 (43.5)	69 (56.1)	
Gestation week	38.6±1.8	38.5±1.9	38.8±1.6	0.076
<37 weeks	41 (12.2)	31 (14.5)	10 (8.1)	0.086
Birth weight, kg	3.0±0.4	3.0±0.5	3.0±0.4	0.077
**Birth order**				0.513
1	245 (72.7)	157 (73.4)	88 (71.5)	
2	67 (19.9)	38 (17.8)	29 (23.6)	
3	23 (6.8)	17 (7.9)	6 (4.9)	
Mother’s age, year	25.5±5.8	25.4 ±5.9	25.6±5.6	0.873
Father’s age, year	29.6±7.5	29.6±7.4	29.6±7.6	0.995
Mother’s educational level*				0.073
Primary or below	4 (1.2)	3 (1.5)	1 (0.8)	
Secondary	315 (97.8)	197 (98.5)	118 (96.7)	
Tertiary or above	3 (0.9)	0	3 (2.5)	
Father’s educational level*				0.104
Primary or below	14 (5.1)	11 (6.5)	3 (2.8)	
Secondary	256 (92.4)	155 (92.3)	101 (92.7)	
Tertiary or above	7 (2.5)	2 (1.2)	5 (4.6)	
Mother’s employment status*	76 (22.8)	49 (23.2)	27 (22.0)	0.789
Father’s employment status*	253 (85.2)	150 (82.9)	103 (88.8)	0.161
**Marital status**				0.980
Unmarried	189 (56.1)	120 (56.1)	69 (56.1)	
Married	139 (41.2)	88 (41.1)	51 (41.5)	
Divorced	9 (2.7)	6 (2.8)	3 (2.4)	
Marital discord	68 (20.2)	41 (19.2)	27 (22.0)	0.539
Domestic violence	18 (5.3)	10 (4.7)	8 (6.5)	0.472
Maternal active smoking during pregnancy*	148 (44.6)	94 (45.0)	54 (43.9)	0.849
Maternal active drinking during pregnancy*	0	0	0	
Maternal psychiatric illness	123 (36.5)	77 (36.0)	46 (37.4)	0.795
Maternal history of chronic illness requiring medical follow up	8 (2.4)	4 (1.9)	4 (3.3)	0.470
**No. of kinds of maternal drug use**				0.142
1	205 (60.8)	123 (57.5)	82 (66.7)	
2	84 (24.9)	55 (25.7)	29 (23.6)	
3	28 (8.3)	23 (10.7)	5 (4.1)	
>3	7 (2.1)	13 (6.1)	7 (5.7)	
**Illicit drug use**				
Amphetamine	183 (54.3)	116 (54.2)	67 (54.5)	0.962
Ketamine	171 (50.7)	111 (51.9)	60 (48.8)	0.585
Cocaine	71 (21.1)	40 (18.7)	31 (25.2)	0.158
Duration of illicit drug use	4.2±3.3	4.0±3.1	4.7±3.6	0.043
Paternal psychiatric illness	8 (2.4)	6 (2.8)	2 (1.6)	0.715
Paternal history of substance abuse	113 (33.5)	64 (29.9)	49 (39.8)	0.063
Comprehensive Social Security Assistance as financial support	118 (35.0)	79 (36.9)	39 (31.7)	0.335
Social support (perceived as good)	294 (87.2)	183 (85.5)	111 (90.2)	0.210
Involvement of other adults in childcaring	235 (69.7)	163 (76.2)	72 (58.5)	<0.001
**Caring adults**				<0.001
Parent(s) only	100 (29.7)	50 (23.4)	50 (40.7)	
Parent(s) + others	28 (8.3)	10 (4.7)	18 (14.6)	
Grandparents only	42 (12.5)	33 (15.4)	9 (7.3)	
Grandparents + others	123 (36.5)	84 (39.3)	39 (31.7)	
Foster	19 (5.6)	18 (8.4)	1 (0.8)	
Residential care	9 (2.7)	8 (3.7)	1 (0.8)	
Others/relatives	16 (4.7)	11 (5.1)	5 (4.1)	
Ever out of home care	38 (11.3)	31 (14.5)	7 (5.7)	0.014
Early schooling (school enrollment at 2 years old)*	51 (23.6)	31 (22.6)	20 (25.3)	0.654
Child with chronic illness requiring follow up	27 (8.0)	16 (7.5)	11 (8.9)	0.633
Age at last follow up, month	37.5±16.0	38.0±16.0	36.6±16.1	0.456

*P*-values derived from Pearson’s chi-square test, Fisher’s exact test, or independent *t*-test.

*With missing data.

205 Mothers had used one kind of illicit drug (60.8%), while 84 (24.9%) used two kinds and 35 (10.4%) used three or more kinds. The most common illicit drug used was amphetamine (54.3%) followed by ketamine (50.7%) and cocaine (21.1%). About 123/337 (36.4%) mothers had a history of psychiatric illness. Adjustment disorder (50.4%), acute psychosis (26.8%), and depression (16.3%) were the top three diagnoses.

No significant congenital malformations were detected in the whole cohort. 95 Children (28.1%) were at the age greater than 4 and 33 (9.8%) were greater than 5 years old at last follow-up and there were no significant differences between the number of children in different age ranges (< = 12 m; < = 18 m; < = 24 m; < = 30 m; < = 36 m; < = 48 m; < = 60 m and > 60 m) at last follow-up between the two groups. The CCDS group had significantly more LBW children (15.9 vs. 7.3%, *p* = 0.023) than the integrated program group, but there were no significant differences in gestation age or SGA incidence. Eleven (3.3%) of the whole cohort had childhood growth problems and only two (0.9%) children had delayed their vaccination for 3 months. Moreover, there were no statistically significant differences for ASD, ADHD, dyslexia, non-specific behavioral and emotional problems, and child abuse incidence between the two groups (Table 2).

**TABLE 2:** Children and maternal outcomes.

	Total	CCDS	Integrated	*P*-value
	(*n* = 337)	(*n* = 214)	(*n* = 123)	
**Children outcomes**				
Low birth weight	43 (12.8)	34 (15.9)	9 (7.3)	0.023
Small for gestational age	24 (7.1)	17 (7.9)	7 (5.7)	0.439
Childhood period: growth problem (fall off 2 centiles)	11 (3.3)	6 (2.8)	5 (4.1)	0.538
Schedule of vaccination – completed/delayed for 3 months	2 (0.6)	2 (0.9)	0	0.535
Specific learning disorder (e.g., dyslexia)	4 (1.2)	1 (0.5)	3 (2.4)	0.140
Attention- Deficit Hyperactive Disorder	15 (4.5)	12 (5.6)	3 (2.4)	0.175
Autistic Spectrum Disorder	11 (3.3)	6 (2.8)	5 (4.1)	0.538
Non-specific behavioral problem	29 (8.6)	22 (10.3)	7 (5.7)	0.148
Emotional problem	16 (4.7)	11 (5.1)	5 (4.1)	0.655
Child abuse/neglect	36 (10.7)	21 (9.8)	15 (12.2)	0.496
Urine positive at newborn	23 (6.8)	12 (5.6)	11 (8.9)	
Urine positive at newborn + other time during follow up	3 (0.9)	1 (0.5)	2 (1.6)	
Urine positive at other time during follow up	4 (1.2)	4 (1.9)	0 (0.0)	
At risk	4 (1.2)	3 (1.4)	1 (0.8)	
Neglect (physical)	2 (0.6)	1 (0.5)	1 (0.8)	
Cognitive delay	47 (13.9)	40 (18.7)	7 (5.7)	<0.001
Language delay	124 (36.8)	94 (43.9)	30 (24.4)	<0.001
Motor delay	58 (17.2)	49 (22.9)	9 (7.3)	<0.001
Social delay	52 (15.4)	43 (20.1)	9 (7.3)	0.002
Global delay	44 (13.1)	37 (17.3)	7 (5.7)	0.002
Any delay in cognitive, language, motor, and social aspects	129 (38.3)	98 (45.8)	31 (25.2)	<0.001
1	68 (20.2)	47 (22.0)	21 (17.1)	
2	14 (4.2)	11 (5.1)	3 (2.4)	
3	3 (0.9)	3 (1.4)	0	
4 (Global delay)	44 (13.1)	37 (17.3)	7 (5.7)	
**Maternal outcomes**				
Maternal abstinence from drug use during pregnancy	244 (72.4)	152 (71.0)	92 (74.8)	0.456
Maternal relapse of drug use after birth*	45 (13.6)	18 (8.7)	27 (22.0)	<0.001
Maternal abstinence from drug use up to 2 years post-delivery*	160 (54.8)	102 (55.1)	58 (54.2)	0.878

*P*-values derived from Pearson’s chi-square test or Fisher’s exact test.

* With missing data.

**FIGURE 1:**
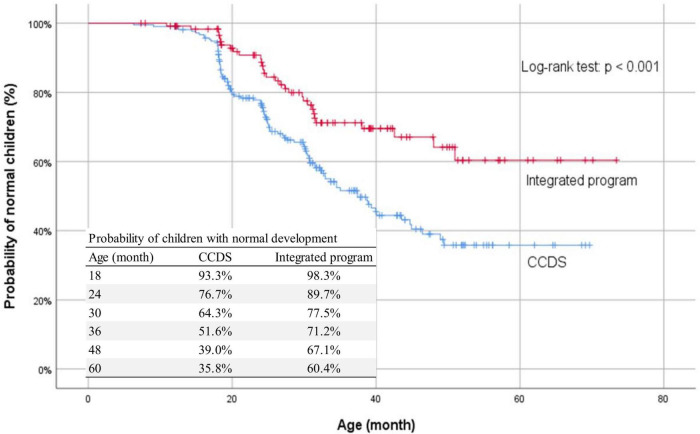
Kaplan-Meier curve on any developmental delay for CCDS and integrated program.

Among the whole cohort (*N* = 337), any developmental delay was present in 129 children (38.3%). The most common delay was language delay which was found in 124 children (36.8%). Global delay was present in 44 (13.1%). Children in the CCDS group had significantly more cognitive delay (18.7 vs. 5.7%, *p* < 0.001), language delay (43.9 vs. 24.4%, *p* < 0.001), motor delay (22.9 vs. 7.3%, *p* < 0.001), social delay (20.1 vs. 7.1%, *p* = 0.002), and global delay (17.3 vs. 5.7%, *p* = 0.002) than children in the integrated program group (Table 2). The probability of children with normal development in two groups was shown in the Kaplan–Meier curve (Figure 1) and their differences by the log-rank test were statistically significant (*p* < 0.001).

No statistically significant differences were found between the two groups regarding maternal abstinence from drug use during pregnancy and up to 2-year post-delivery, but there was statistically more maternal relapse after birth in the integrated program group than in the CCDS group (*p* < 0.001) (Table 2).

Univariable Cox regression analysis was performed for all socioeconomic factors and participation in the integrated program to find out which factors were significantly associated with any developmental delay (Table 3A). The significant factors with hazard ratio less than 1 were integrated program (0.49, 95% C.I. 0.33–0.73), birth order being 1 (0.67, 95% C.I. 0.47–0.97), social support (0.36, 95% C.I. 0.23–0.54), and maternal abstinence from drug use up to 2-years post-delivery (0.60, 95% C.I. 0.42–0.87). The significant factors with hazard ratio greater than 1 were male gender (2.00, 95% C.I. 1.38–2.88), CSSA (1.51, 95% C.I. 1.07–2.14), and foster/residential placement (2.28, 95% C.I. 1.34–3.89). Factors with *p* < 0.1 in the univariable analysis were further assessed using multivariable Cox regression analysis (Table 3B). The factors with an adjusted hazard ratio of less than one were integrated program (0.53, 95% C.I. 0.34–0.84), social support other than CCDS or integrated program (0.45, 95% C.I. 0.25–0.80), and maternal abstinence from drug use up to 2-year post-delivery (0.62, 95% C.I. 0.40–0.95). Male gender (1.73, 95% C.I. 1.18–2.54) was the significant factor with an adjusted hazard ratio greater than 1 were found by the Cox regression multivariable analysis (Table 3B). Maternal abstinence from drug use up to 2-year post-delivery was found to be associated with less child neglect (*p* < 0.001) and non-specific behavioral problems (*p* = 0.011) significantly (Table 4).

**TABLE 3A:** Factor associated with any developmental delay: Cox regression univariable analysis.

	HR_*unadj*_ (95% CI)	*P*
Integrated program	0.49 (0.33–0.73)	<0.001
Child male gender	2.00 (1.38–2.88)	<0.001
Preterm, Gestation < 37 weeks	1.06 (0.64–1.74)	0.825
Birth order: 1	0.67 (0.47–0.97)	0.036
Mother’s age < 18 years	0.45 (0.11–1.83)	0.265
Mother’s educational level: Primary or below	1.11 (0.28–4.51)	0.879
Father’s educational level: Primary or below	1.65 (0.80–3.41)	0.173
Mother’s employment status	0.98 (0.65–1.50)	0.939
Father’s employment status	0.74 (0.46–1.17)	0.197
Marital status (Unmarried/divorced vs. married)	1.25 (0.87–1.80)	0.220
Marital discord	1.20 (0.80–1.81)	0.381
Domestic violence	1.64 (0.86–3.12)	0.135
Maternal smoking	0.95 (0.67–1.35)	0.777
Maternal psychiatric illness	1.22 (0.86–1.74)	0.256
Maternal history of chronic illness requiring medical follow up	0.62 (0.15–2.53)	0.510
No. of maternal drug use > 3	0.83 (0.39–1.77)	0.623
Maternal Amphetamine use	1.26 (0.89–1.80)	0.198
Duration of maternal illicit drug use	1.04 (0.98–1.10)	0.235
Paternal psychiatric illness	1.25 (0.46–3.39)	0.659
Paternal history of substance abuse	0.73 (0.50–1.06)	0.098
Comprehensive Social Security Assistance as financial support	1.51 (1.07–2.14)	0.019
Social support	0.36 (0.23–0.54)	<0.001
Caring adults		
Parent(s) (ref.)	1	–
Grandparents/relatives	0.97 (0.66–1.43)	0.875
Foster/residential care	2.28 (1.34–3.89)	0.002
Maternal abstinence from drug use up to 2 years post-delivery	0.60 (0.42–0.87)	0.007

**TABLE 3B:** Factor associated with any developmental delay: Cox regression multivariable analysis.

	HR_*adj*_ (95% CI)	*P*
Integrated program	0.53 (0.34–0.84)	0.006
Child male gender	1.73 (1.18–2.54)	0.005
Birth order: 1	1.02 (0.66–1.56)	0.941
Paternal history of substance abuse	0.77 (0.52–1.15)	0.203
Comprehensive Social Security Assistance as financial support	1.02 (0.67–1.54)	0.942
Social support	0.45 (0.25–0.80)	0.006
Caring adults		
Parent(s) (ref.)	1	–
Grandparents/relatives	0.88 (0.55–1.41)	0.589
Foster/residential care	0.83 (0.41–1.65)	0.586
Maternal abstinence from drug use up to 2 years post-delivery	0.62 (0.40–0.95)	0.030

*Includes variables with *p* < 0.1 in the univariable analyses.

**TABLE 4:** Children outcomes in relation to maternal abstinence from drug use for up to 2-year post-delivery.

	Maternal abstinence from drug use up to 2 years post-delivery*		*P*-value
		
	No	Yes	
Child abuse/neglect	34 (25.8)	1 (0.6)	<0.001
Non-specific behavioral problem	19 (14.4)	9 (5.6)	0.011
Emotional problem	9 (6.8)	7 (4.4)	0.361

*P*-values derived from Pearson’s chi-square test.

*Missing data.

## Discussion

### Maternal illicit drug use and childhood adverse outcomes

The incidence of preterm delivery was 12.2% in the cohort which was higher than the reported frequency of 6.5% among the singleton deliveries in one local study (37) but lower than the reported 33.3% in the heroin cohort when CCDS was not introduced (38). Maternal illicit drug use (39) and amphetamine abuse (40, 41) were reported to be associated with higher risk of preterm delivery and LBW. Moreover, smoking during pregnancy was associated with LBW and intrauterine growth restriction (2, 42). LBW was also related to maternal low socioeconomic class (43). The incidence of LBW in the cohort was 12.8% which was higher than 6.44% as reported in a local teaching hospital (44). The CCDS group had significantly higher LBW than the integrated group (15.9 vs. 7.3%), but there were no significant differences in maternal amphetamine use, smoking, and socioeconomic status between the two groups, so the difference could be explained by maternal participation in the integrated program. It had been reported to decrease preterm delivery as women could receive prenatal care more consistently in the integrated program (45). Expectant mothers were engaged early in pregnancy, the CCDS midwife (case manager) provided antenatal nutritional advice and healthcare, and close liaison of HKLSS social workers facilitated regular antenatal attendance, resulting in a lower incidence of preterm birth (8.1 vs. 14.5%) in the integrated program group, though this was not statistically significant. The abstinence from drug use as counseled by HKLSS social worker (confirmed by urine testing) and supported by the team members enabled mothers to a better nutritional status (46) and could account for the lower incidence of LBW in the integrated group.

The incidences of having childhood growth problems were low in both groups as the children’s growth and vaccination schedule were continuously monitored and managed by CCDS pediatrician. Only two children had their vaccinations delayed for more than 3 months for insignificant reasons. This result was promising as only 77% of children born to heroin-abusing mothers completed the recommended vaccination schedule in pre-CCDS era (38).

Various developmental delays, behavioral and emotional problems were detected in the study which was also reported in previous literature (10–16, 18–23, 28–30). We detected a significant proportion (38.3%) of children born to drug-abusing mothers with any kind of developmental delay; the most common type was language delay. The children’s early years of development are closely determined by the interrelationship with the caretakers’ provision of secure attachment and the environmental stimulation (47). These families came from complex social backgrounds, lower socioeconomic status, and 36.4% of recruited mothers also had a history of mental illness; although most mothers stopped using drugs during pregnancy, they were not able to provide good-enough parenting without comprehensive support. As a result, their children could not receive optimal stimulation or responsive parenting to promote normal development. An integrated program provides emotional support to mothers through counseling, trust building with the CCDS team and HLKSS social workers with improved medical compliance, practical childcare support through doula and social worker home visits, detoxification support through peer groups, and parenting coaching through group activities. Regular case review meetings by the CCDS team and HKLSS social workers allowed timely intervention on handling maternal drugs, respective family issues, childcare, and parenting problems. All these factors contributed to build up a more stable environment and maternal reflective capacity for nurturing the child’s growth and development. This was illustrated by the significant differences in the various types of developmental delay between the two groups as shown in the Kaplan–Meier curve.

The incidences of ADHD, behavioral, and emotional problems in the entire cohort were relatively low, which may be due to (1) most pregnant mothers discontinuing illicit drug use in pregnancy, as continuous use of amphetamine throughout gestation was associated with a higher prevalence of ADHD, aggression, and learning difficulties (48) and (2) insufficient length of follow-up, with only 10% of the cohort aged 5 or older at the last follow-up. Attention control and impulse control problems have been reported in school children up to 10-year-old born to cocaine-abusing mothers (49), implying that these developmental problems will become more apparent as the child grows older, allowing a definite diagnosis to be made.

Three-fold increase in the risk of child maltreatment was reported in the children of drug-abusing parents (50); however, in this cohort, the majority of the cases were determined to be child neglect because the child tested positive for urine in a short period of time after delivery (23 out of 36, i.e., 6.8 vs. 10.7% of the total cohort). Saving newborn urine for toxicology has been a common practice in KEC since 2016 if expectant mothers are active drug users. Only two cases were detected as child physical neglect in their childhood period. This study found that childcare surveillance, parenting support, and involvement of other stable caretakers in both groups could significantly reduce child abuse in this high-risk population.

### Maternal outcomes

About 72% of expectant mothers abstained from illicit drug use in the cohort and 54.8% of them remained drug-free up to 2-year post-delivery. The one-stop service of CCDS allowed accessibility for healthcare and the non-judgmental attitude of the CCDS team and social workers established a trusting relationship with mothers, promoting family and social support to mothers’ motivation for sustaining detoxification. However, the higher relapse rate in the integrated group could be due to a more precise method of detection, i.e., by urine testing and frequent social encounters by HKLSS workers to detect maternal behaviors of drug relapse.

### Associated factors on any developmental delay

As discussed above, the integrated program adopted a bio-psycho-social approach and used pregnancy as a golden window of opportunity to engage illicit drug-abusing mothers and provided the necessary intervention; thus, similar effects on reducing developmental adverse outcomes had been reported in the literature (51). Childcare for the integrated program group was mostly provided by their mothers. With the sustainability of drug-free up to 2-year post-delivery enabled the mother to adopt a normal lifestyle and emotional stability, which were essential factors for good reflective functioning to tune to child’s emotional and developmental needs for better developmental outcomes, less behavioral problems, and child abuse. A parent’s capacity for reflective function has a positive impact on early mother–infant interaction, child attachment, and development (52, 53). Early experiences with parent/caretaker are crucial for child’s cognitive, emotional, and social development (47).

Good social support in the form of supportive relationship with family members, partners, and friends has been shown to be beneficial in assisting substance users in maintaining drug abstinence (54, 55). Social support provided means of childcare and possibly offered appropriate stimulation for the children’s optimal development.

About 5 to 12% of preschool children have language delay (56), while 13.5–17.5% of children aged 18–36 months are reported to have expressive language delay (57). Male is the predominant gender and it was consistent with previous literature findings (58, 59). The delay in speech was related to a lack of quality stimulation and poor linguistic home environment in these drug-abusing families.

Socioeconomic factors have been reported to be related to the developmental outcomes of these high-risk children (23–25) but we did not find any significant associative effect which may be related to the small subsample size within each individual social factor for both parents, particularly for fathers.

### Implications of the findings

Detoxification program should be coupled with parenting coaching and multidisciplinary coordination is needed to engage drug-abusing expectant mothers to retain in the program to improve the growth and developmental outcomes of their children. Setting up of a community-based early training center is needed to promote quality stimulation for development, particularly for speech to this group of vulnerable children. Long-term follow-up is warranted to have a more comprehensive picture of the developmental trajectory of these children because compromised neurobehavioral outcomes may be evident only at school age or adolescence.

### Public health implications

Coordinated multidisciplinary collaboration with local resources to ensure continuity of care to drug-abusing mothers and their children was well demonstrated. A caring relationship with service providers as means of support for detoxification is recognized as helpful for substance use disorder recovery (55, 60). The integrated program enhances maternal parenting capacity for optimal childhood development; and reduces the risk of child maltreatment and hence possibly less adolescent mood and drug problems in their offspring (61) which can break the intergenerational cycle of drug abuse. This may reduce the public costs in tackling the subsequent medical and psychosocial problems.

### Strength and limitations

This was the first local study on the integrated intervention to a Chinese cohort of illicit soft drug-abusing parents and their children. The study was a longitudinal one with frequent assessments of clinical cases and their diagnoses were made by experienced professionals. Comprehensive child adverse outcomes and potential associative factors were searched. The study’s retrospective nature limited its scope; data may be incomplete, and cofounders may not have been identified. Self-reported drug use by mothers may be inaccurate and social support was a subjective measure rather than using a validated assessment scale. Moreover, not every case has been followed up to 5 years or above which could lead to lower incidence of behavioral, emotional, and learning problems.

## Conclusion

It is salient to provide services equitable and accessible to the drug-abusing expectant mothers and their children to obtain optimal health gain for both mother and child. CCDS achieves early engagement of these vulnerable families during pregnancy; both CCDS and the Integrated Program can improve vaccination rates, detect growth problems in children early, provide parenting support, minimize child abuse, and reduce maternal risk behaviors. With an integrated program involving multidisciplinary collaboration, the value of the service is increased by lowering the incidence of low birth weight and being able to sustain mothers’ abstinence from drug use up to 2-year post-delivery using more targeted detoxification intervention and objective measurements (urine testing), regular review as means of timely communication between CCDS team members and HKLSS social workers, allowing mothers to adopt a better lifestyle and emotional stability to nurture and hence reduce the developmental adverse outcomes in these children and improve their school readiness. Resources should be allocated to promote the integrated program across the territory of HKSAR to achieve positive impacts on these disadvantaged children. Future research in multiple sites with different ethnic women is needed to study the generalizability of the integrated program in these vulnerable mothers and children.

## Data availability statement

The availability of data analyzed in this study is subject to the approval of the Kowloon Central Ethics Committee, Hospital Authority. Requests to access these dataset can be directed to the corresponding author.

## Ethics statement

The studies involving human participants were reviewed and approved by the Kowloon Central Cluster Ethics Committee, Hong Kong Hospital Authority. Written informed consent from the participants’ legal guardian/next of kin was not required to participate in this study in accordance with the national legislation and the institutional requirements.

## Author contributions

AC conceptualized and designed the study, set up the CCDS and integrated program, was involved in the care of the children and mothers, collected, and analyzed the clinical information and data, drafted the initial manuscript, and revised the manuscript. LI and KW were involved in the care and assessment of the follow-up mothers, collected clinical information and data, and reviewed the manuscript. HC and WC supervised the CCDS and integrated program and critically reviewed the manuscript. EYu conceptualized clinical information and data, was involved in the data analysis, and reviewed the manuscript. PC and EYe critically reviewed the manuscript for important intellectual content. All authors approved the final manuscript as submitted and agreed to be accountable for the work.
