# Supplementary material for: The physical and developmental outcomes of children whose mothers are substance abusers: Analysis of associated factors and the impact of early intervention
Source: Front Pediatr. 2022 Oct 20;10:1004890. doi: 10.3389/fped.2022.1004890 (PMC9631827; doi:10.3389/fped.2022.1004890)
Supplement: Supplementary file 1 [file Data_Sheet_1.pdf]

Sample size calculation (Supplementary file)

Assuming there was 18% of children had developmental delay in CCDS group (p_0) and 8% of children had developmental delay in the integrated program (p_1). To give the study power of 80% to detect at alpha =0.05 (2- sided level) and according to Kelsey formula

$$N_{Kelsey} = \frac{(Z_{\alpha/2} + Z_{\beta})^2 \, p\,(1 - p)\,(r + 1)}{r\,(p_0 - p_1)^2}$$

$$p = \frac{p_0 + rp_1}{r + 1}$$

p_0: The CCDS cohort

p_{1}: The Integrated Program Cohort

r: the ratio of exposed (p_1: integrated) to nonexposed (p_0: CCDS) = 1: 1.5

Sample size needed :139 for CCDS ;139/1.5= 93 for integrated programme
